# Macrophages From Subjects With Isolated GH/IGF-I Deficiency Due to a GHRH Receptor Gene Mutation Are Less Prone to Infection by *Leishmania amazonensis*

**DOI:** 10.3389/fcimb.2019.00311

**Published:** 2019-08-30

**Authors:** Mônica R. Barrios, Viviane C. Campos, Nalu T. A. Peres, Laís L. de Oliveira, Rodrigo A. Cazzaniga, Márcio B. Santos, Murilo B. Aires, Ricardo L. L. Silva, Aline Barreto, Hiro Goto, Roque P. Almeida, Roberto Salvatori, Manuel H. Aguiar-Oliveira, Amélia M. R. Jesus

**Affiliations:** ^1^Division of Immunology and Molecular Biology Laboratory, Federal University of Sergipe, Aracaju, Brazil; ^2^Division of Endocrinology, Federal University of Sergipe, Aracaju, Brazil; ^3^Department of Microbiology, Institute of Biological Sciences, Federal University of Minas Gerais, Belo Horizonte, Brazil; ^4^Laboratório de Soroepidemiologia e Imunobiologia, Instituto de Medicina Tropical de São Paulo, Universidade de São Paulo, São Paulo, Brazil; ^5^Division of Endocrinology, Diabetes and Metabolism, The Johns Hopkins University School of Medicine, Baltimore, MD, United States

**Keywords:** growth hormone deficiency, insulin growth factor-I deficiency, macrophages, leishmaniasis, phagocytosis, infection, immunology

## Abstract

Isolated growth hormone (GH) deficiency (IGHD) affects approximately 1 in 4,000 to 1 in 10,000 individuals worldwide. We have previously described a large cohort of subjects with IGHD due to a homozygous mutation in the GH releasing hormone (GHRH) receptor gene. These subjects exhibit throughout the life very low levels of GH and its principal mediator, the Insulin Growth Factor-I (IGF-I). The facilitating role of IGF-I in the infection of mouse macrophages by different *Leishmania* strains is well-known. Nevertheless, the role of IGF-I in *Leishmania* infection of human macrophages has not been studied. This study aimed to evaluate the behavior of *Leishmania* infection *in vitro* in macrophages from untreated IGHD subjects. To this end, blood samples were collected from 14 IGHD individuals and 14 age and sex-matched healthy controls. Monocytes were isolated and derived into macrophages and infected with a strain of *Leishmania amazonensis*. In addition, IGF-I was added to culture medium to evaluate its effect on the infection. Cytokines were measured in the culture supernatants. We found that macrophages from IGHD subjects were less prone to *Leishmania* infection compared to GH sufficient controls. Both inflammatory and anti-inflammatory cytokines increase only in the supernatants of the control macrophages. Addition of IGF-I to the culture medium increased infection rates. In conclusion, we demonstrated that IGF-I is crucial for *Leishmania* infection of human macrophages.

## Introduction

Growth Hormone (GH) and its peripheral effector Insulin Growth Factor-I (IGF-I) have mitogenic and anabolic actions in various cells, including immune cells. This observation created an interest in understanding the role of GH in the endocrine-immune axis (Wells, 1999). The effect of IGF-I in pathogen-macrophage interaction has been described in *Leishmania* and *M. leprae* infections (Reis et al., 2013; Batista-Silva et al., 2016).

*Leishmania* is an obligate intracellular parasite that infects macrophages. In humans, it causes a broad clinical spectrum of diseases, such as cutaneous, mucosal, and visceral leishmaniasis. This diversity of manifestations depends on the parasite species, environmental, biological, or genetic factors of the host, particularly the immune response (Oryan and Akbari, 2016). The model of infection using *Leishmania* is easy to culture and manipulate, and has been widely used in studies that seek to understand the immune response to intracellular infections (de Oliveira et al., 2015; Silva et al., 2017).

Goto et al. (1998) showed that IGF-I enhanced, *in vitro*, the growth of promastigotes and amastigotes in mouse macrophages (Goto et al., 1998) and that IGF-I-pre-treated *Leishmania* have enhanced infectivity (Gomes et al., 2000). Furthermore, IGF-I increases the expression and activity of arginase I (that promotes the parasite survival), and blocks the induction of NOS2, an enzyme involved in nitric oxide production, essential to kill *Leishmania* (Vendrame et al., 2007, 2015). In addition, recent studies have shown that during infection, *M. leprae* induces the production of IGF-I in both macrophages and Schwan cells, an important mechanism for the survival of this pathogen (Rodrigues et al., 2010; Batista-Silva et al., 2016). These data suggest that IGF-I plays an important role in the survival and proliferation of these intracellular pathogens. The use of hormonal pathways by intracellular pathogens to evade the immune system may be one important mechanism of these pathogens to explain their adaptation to survive in human cells.

In Itabaianinha County, in Northeast Brazil, we identified a large cohort of individuals with severe isolated GH deficiency (IGHD) caused by the *null* homozygous (c.57 + 1 A → G) mutation in the growth hormone releasing hormone (GHRH) receptor gene (*GHRHR*) (Salvatori et al., 1999). This is the largest cohort of patients with IGHD described to date. This mutation leads to a complete abolition of the GHRHR function and consequently affects GH secretion (Souza et al., 2005), leading to very low levels of GH and IGF-I (Aguiar-oliveira et al., 1999; Salvatori et al., 2006). These IGHD individuals have proportional short stature, doll face, high-pitched voices, and central obesity (Aguiar-Oliveira and Bartke, 2018). They have relatively reduced spleen volume (Oliveira et al., 2008), reduced total serum IgG levels, and present smaller papule diameter after streptokinase injection. Despite these abnormalities, they seem to have normal immune function and do not exhibit increased frequency of infections (Campos et al., 2016). Not surprisingly, they exhibit normal longevity, and some reached centenarian age (Aguiar-Oliveira et al., 2010; Aguiar-Oliveira and Bartke, 2018).

Most of the adult IGHD individuals have never been treated with GH replacement therapy, and therefore provide a unique model to evaluate the role of IGF-I on immune cells in a variety of conditions. The purpose of this study was to evaluate the behavior of *Leishmania* infection *in vitro* in macrophages of these IGHD subjects.

## Materials and Methods

### Subjects

Fourteen IGHD individuals with genotype proven homozygosis for the C.57 + 1G > A *GHRHR* mutation, and 14 age and sex-matched normal statured local controls proven to be homozygous for the wild-type *GHRHR* allele were included in this study. The Research Ethical Committee at Federal University of Sergipe approved this study (CAAE 0152.0.107.000-07). After signing informed consent form, the participants were submitted to clinical examination, measurement of height, weight and venipuncture to collect peripheral blood. Two of the IGHD subjects had received GH therapy for 6 years, completed more than 15 years prior to this study.

### Hormones Measurements

IGF-I was measured by a solid-phase, enzyme labeled chemiluminescent immunometric assay IMMULITE 2000 (Siemens Healthcare Diagnostics Products Ltd, Malvern, PA, USA), with a sensitivity of 25 ng/ml. Prolactin was measured by fluoroimmunoassay (Perkin Elmer Life and Analytical Science, Wallac Oy Turku, Finland) with sensitivity of 1.44 ng/ml.

### IGF-I and IGF-I Receptor (IGF-IR) mRNA Expression in PBMC

Peripheral blood mononuclear cells (PBMC) were isolated by Ficoll gradient from whole blood. Total RNA was extracted by Trizol reagents (ThermoFisher), and 1 μg RNA was converted into cDNA using the High Capacity cDNA Synthesis Kit (ThermoFischer), following the manufacturers' protocol. qPCR was performed using Taqman probes (hs01547656_m1– IGF-I and hs00609566_m1 – IGF-IR), in the 7500 Fast Real Time PCR System (Applied Biosystems). Normalization was performed using the GAPDH gene (hs99999905_m1), and relative gene expression was represented by the 2^−Δ*Ct*^ method (Livak and Schmittgen, 2001).

### Macrophage Infection

Macrophage cultures and infection with *Leishmania* were processed according to previous publications (de Oliveira et al., 2015; Silva et al., 2017). Briefly, PBMC were fractioned by Ficoll-Hypaque (Histopaque® 1077, Sigma). The monocytes were isolated by adherence to plastic plates and maintained in culture medium RPMI supplemented (albumin and antibiotics) for 6 days in a humid incubator (37°C and 5%CO^2^) to differentiate into macrophages in LabTek (de Oliveira et al., 2015). *Leishmania amazonensis* (LTCP 9667) were cultured in medium supplemented with Schneider (ThermoFisher) in a dry incubator (26°C) and were used to infect macrophages (5:1) for 2 h in a humid incubator (37°C and 5% CO2). Macrophages from healthy controls (*n* = 6) and IGHD individuals (*n* = 4) were also infected with *Leishmania* without or with IGF-I addition (75 ng/ml) 2 h before infection, or infected with IGF-I pre-treated *Leishmania* (50 ng/ml) for 5 min and washed off with saline supplemented with 1% albumin (ThermoFisher) (*n* = 4), as previously described by Gomes et al. (2000). We measured the number of amastigotes in 100 macrophages at 2 h of *Leishmania* infection. The supernatants were collected and stored at −80°C and macrophages slides were stained after 2, 24, 48, and 72 h post infection. The macrophages slides infected with IGF-I-treated macrophages and *Leishmania* pre-treated with IGF-I were stained 2, 24, and 48 h post infection. Slides were fixed and then stained with Instant Prov (NEW/PROV, Paraná, Brazil). Three blinded scientists counted the number of infected macrophages per 100 macrophages, and the number of amastigotes per 100 infected macrophages.

### Cytokine Measures

The cytokines IL-12p70, TNF-α, IFN-γ, IL-1β, IL-6, IL-10, GM-CSF, IL-4, IL-33, and IL-27 were measured in the macrophage supernatants previously stored at −80°C. Cytokines quantification was done by multiplex assay (Procarta, Thermo, Waltham, MA USA).

### Statistical Analysis

Data analyses were processed in GraphPad Prism Software (v.4.0). Values for parametric variables were expressed as mean (standard deviation). Variables with non-parametric distribution (IGF-I levels) were expressed as median (interquartile range). Gender was compared with the Fisher's exact test. D'Agostino and Pearson and Shapiro–Wilk normality tests were used to verify if the groups follow a normal distribution. Student *t*-test was used for comparisons between two variables with normal distribution and Mann–Whitney *U*-test for those with non-Gaussian distribution, adopting 95% as confidence interval and significance values when *p* < 0.05.

## Results

### Demographic Characteristics and Levels of IGF-I and Prolactin, and IGF-I and IGF-I Receptor mRNA Expression of the Study Subjects

**Figure 1:**
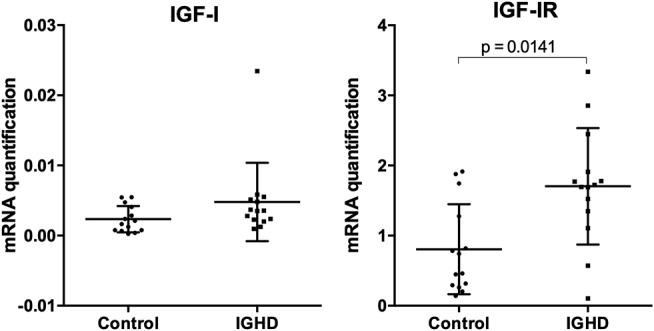
mRNA expression of IGF-I and IGF-I receptor in PBMC of IGHD and control subjects. Total RNA was extracted from PBMC of 14 IGHD individuals and 14 controls, and qPCR was performed using Taqman probes. Normalization was performed using the GAPDH gene, and relative gene expression is represented by the 2^−Δ*Ct*^ method.

Clinical and demographic data, IGF-I and prolactin levels are shown in Table 1. There were no differences in age and sex distribution between IGHD and controls. As expected, height, weight, and IGF-I levels were lower (*p* < 0.0001) in the IGHD group than controls. Most IGF-I levels in IGHD group were lower than the sensitivity of assay, emphasizing the severity of IGF-I deficiency. There was no difference in prolactin levels between the groups (Table 1). No differences in the mRNA expression of IGF-I was observed between IGHD subjects and controls, but higher levels of IGF-1R mRNA was found in PBMC from IGHD subjects as compared to controls (Figure 1).

**Table 1:** Demographic and serum levels of IGF-I and prolactin in subjects with isolated GH deficiency due to a GHRH receptor gene mutation and controls.

**Variables**		**IGHD** **(*n* = 14)**	**Control** **(*n* = 14)**	***P***
Age (years)	Mean (SD)	40.4 (10.7)	41.3 (14.3)	0.84
	Range	27–58	21–61	
Male		09 (60%)	08 (50%)	0.58
Height (cm)	Mean (SD)	131.8 (8.1)	168.9 (7.7)	< 0.0001
	Range	119–143	157–187	
Weight (kg)	Mean (SD)	38.5 (7.1)	73.5 (13.1)	< 0.0001
	Range	27.4–51.7	52.5–105	
Serum IGF-I (ng/ml)	Median (Interquartile)	25 (0)	170 (55)	< 0.0001
	Range	25–52.8	104–340	
Serum prolactin (ng/ml)	Mean (SD)	15.2 (7.9)	11.8 (5.9)	0.38
	Range	2.07–23.4	3.7–26.3	

*Age, height, weight, and prolactin were compared by the unpaired T-Test; gender by the Fisher's Exact Test; and IGF-I by the Mann–Whitney Test*.

### Macrophages Infection

**Figure 2:**
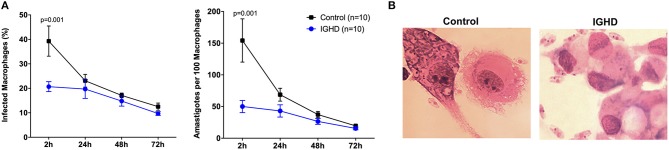
*Leishmania amazonensis* infection curves in human macrophages from IGHD subjects and controls. **(A)** Number of macrophages infected with *L. amazonensis* in 100 macrophages and number of amastigotes in 100 macrophages from IGHD patients and healthy controls. **(B)** Photomicrography of 2 h-infected macrophages from controls subjects and IGHD subjects.

The infection curve of *L. amazonensis* in macrophages from IGHD subjects and controls demonstrate both a lower number of infected macrophages and parasitic load (number of amastigotes per 100 infected macrophages) in IGHD group, in the early hours of exposure to the parasite (*p* < 0.05) (Figure 2A). Figure 2B is a Photomicrography of 2 h-infected macrophages from controls subjects and IGHD subjects showing an example of the differences between the infection in these two hosts. While macrophage from the control subjects have many parasites inside the cytosol, the macrophages from IGHD subjects have parasites around the membrane and very few parasites inside the cytosol.

### Cytokine Levels

**Figure 3:**
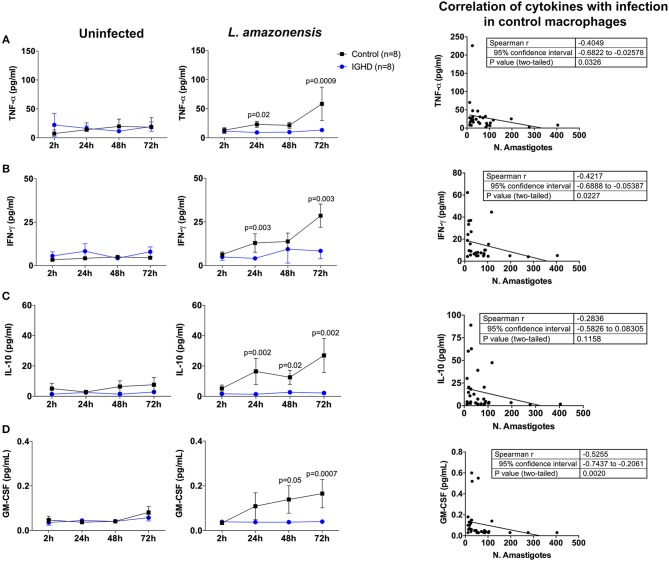
Cytokines produced by human macrophages from IGHD subjects and controls during *in vitro Leishmania amazonensis* infection. The levels of the cytokine were measured by luminex in the culture supernatants, and correlation with infection determined in control macrophages. **(A)** TNF-α, **(B)** IFN-γ, **(C)** IL-10, and **(D)** GM-CSF.

**Figure 4:**
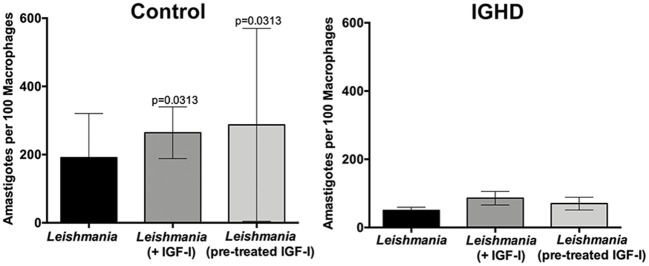
*Leishmania amazonensis* infection in human macrophages from IGHD subjects and controls, in the absence or presence of IGF-I. Infection of macrophages from healthy controls (*n* = 6) and IGHD subjects (*n* = 4) without or with IGF-I addition (75 ng/ml) 2 h before infection, or infected with pre-treated leishmania (50 ng/ml) for 5 min and washed off with saline supplemented with 1% albumin (*n* = 4). The graphs show the number of amastigotes in 100 macrophages at 2 h of *Leishmania* infection.

The levels of cytokines in the supernatants of these cultures revealed a significant increase in TNF-α (Figure 3A), IFN-γ (Figure 3B), IL-10 (Figure 3C), and GM-CSF (Figure 3D) only in the control group after 24 h of *L. amazonensis* infection, with a gradual increase until 72 h post-infection, as compared to the IGHD subjects (*p* < 0.05). An inverse correlation was observed between the levels of TNF-α (rS = 0.40, *p* = 0.03), IFN-γ (rS = 0.42, *p* = 0.02), and GM-CSF (rS = 0.52, *p* = 0.002) with the parasite numbers. No correlation was observed between the levels of IL-10 with the number of parasites. No differences were observed in the levels of IL-12p70, IL-1β, IL-6, IL-4, IL-33, IL-27 either during the time-points of infection or between the two groups. Addition of IGF-I to the macrophage cultures, or pre-treatment of the leishmania with IGF-1 increased *L. amazonensis* infection in control macrophages at 2 h of the infection (Figure 4), but not at 24 and 48 h (data not shown). However, this treatment did not affect the infection in macrophages from the IGHD subjects (Figure 4).

## Discussion

Our study shows that macrophages of IGHD individuals with very low serum IGF-I levels present lower levels of infection by *L. amazonensis*, as compared to control macrophages. We hypothesize that IGHD subjects have reduced phagocytic uptake of *Leishmania*, or possibly reduced phagocytic entry of this parasite into the macrophages. This, together with the demonstration that pre-treatment of *Leishmania* with IGF-I increases macrophage infection of control macrophages, is the first report of a prominent role of IGF-I in *Leishmania* infection in humans. These data agree with previous studies in mouse. In this animal, Goto et al. have shown that IGF-I may be used by *Leishmania* as a growth factor (Gomes et al., 1997, 1998; Goto et al., 1998). In addition, IGF-I increases, *in vitro*, the infectivity of this parasite in mouse macrophages (Vendrame et al., 2007, 2015), and *in vivo*, in experimental models of cutaneous leishmaniasis, with strains of *Leishmania mexicana* and *L. amazonensis* (Gomes et al., 2000).

Due to the high homology between GH and prolactin receptors, and the possibility of a cross talk between the two ligands and receptors (Fu et al., 1992), we decided to include prolactin measurements in this protocol. The lack of difference in serum prolactin in the two groups exclude any role of this hormone in reduction of L*. amazonensis* uptake by the macrophages of IGHD subjects.

IGF-IR is expressed in 97% of monocytes, 88% of B lymphocytes and 2% of T lymphocytes (Schwartz et al., 1993; Oberlin et al., 2009). Activation of this receptor leads to stimulation of cell proliferation and differentiation, angiogenesis, and apoptosis inhibition (Juul, 2003). IGF-I also promotes increased expansion of granulocyte and macrophage colonies (Schwartz et al., 1993). Moreover, autocrine production of IGF-I in cells such as macrophages acting on innate immunity indicates a role of IGF-I in modulating the immune response and phagocytosis (Oberlin et al., 2009).

The previously published proposed mechanism to explain the increase of parasite load by IGF-I includes the observation that this hormone increases arginase activity and the production of urea and L-ornithine, nutrients for parasite growth, and inhibits the NOS2 pathway and the production of nitric oxide (NO) (De Souza et al., 2016). It is possible that IGF-I is used as an adaptation mechanism, developed by *Leishmania* to counteract the immune system and to establish the infection. Another finding that reinforces the hypothesis of a role of IGF-I in favoring infection is the presence of a receptor antigenically similar to the human IGF-I receptor (IGF-IR) alpha chain in the promastigote forms of *Leishmania* (Gomes et al., 2001).

Here we demonstrate reduced uptake of *Leishmania* from IGHD macrophages. Interestingly, the addition of IGF-I to the macrophages culture media during infection, or the pre-treatment of *L. amazonensis* with IGF-I, increased the parasite load at the initial stages of infection only in the control group, suggesting that IGF-I favors *Leishmania* infection. We cannot explain why the addition of IGF-I in the macrophages from the IGHD subjects did not have a similar effect, although we observed a higher level of IGF-IR mRNA in PBMC from IGHD subjects, compared to controls. More experiments will be needed to establish if this difference in the expression of this receptor is also confirmed on the macrophage surface. Given that the macrophages from IGHD subjects are able to express IGF-IR, a possible explanation is that the chronic absence of the IGF-I stimuli in these subjects throughout their lives might decrease its activation pathway.

The conditions that can affect the macrophages polarization and their implication on infectious diseases remain unclear. It is believed that this gap can be the key to understand several infectious diseases (Sridharan et al., 2015). The production of IGF-I and IGF-IR by the macrophages suggest an auto/paracrine function on these cells. Furthermore, it has been shown that IGF-I favors a M2 differentiation (Barrett et al., 2015). Recent studies have shown that *M. leprae* infection *in vitro* induces IGF-I production by macrophages and Schwan cells, favoring the establishment of the infection in these cells (Rodrigues et al., 2010; Batista-Silva et al., 2016).

The cytokine profile showed that both inflammatory and anti-inflammatory cytokines increases only in the control macrophages supernatants, concomitantly with the decrease of parasite load. Macrophages from individuals with IGHD were not infected by *Leishmania*, nor did they produce any of these cytokines in their supernatants. These data do not corroborate the hypothesis that the IGHD subjects, who are not exposed to the effects of IGF-I, present macrophages with increased inflammatory and microbicidal ability. Additional studies should be performed to clarify the mechanism of the reduced uptake of *Leishmania* by the macrophages from IGHD subjects.

Our hypothesis is that individuals with IGHD have a reduced phagocytic or non-phagocytic uptake of *Leishmania*. The clinical relevance of these findings still needs to be clarified. One limitation of this study is its descriptive nature, as we could not identify a clear mechanism that explains our findings in IGHD subjects. While we did not find differences in the measured cytokines between IGHD and controls, many other additional cytokines and other mediators, independent of IGF-I, could be analyzed which could, in future studies, shed a light on the molecular mechanism of the identified resistance. As the State of Sergipe is endemic for visceral leishmaniasis, but not for cutaneous forms, a protective role against *Leishmania* infection may have reduced the frequency of the visceral endemic form in the IGHD group. In agreement, during the 25-year follow-up of this cohort, no case of cutaneous or visceral leishmaniasis was recorded (Campos et al., 2016). In addition, the lower uptake of this and possibly other intracellular infectious agents may have contributed to the survival of individuals with IGHD over many generations, and to the spread of this particular mutation in this tropical area. Interestingly, Tripanosomiasis was recorded in normal homozygous controls, but not in IGHD subjects even if they have lived at the same address for decades (Campos et al., 2016).

In summary, this study demonstrates that a unique population of subjects that are genetically deficient in GH/IGF-I that present a reduced uptake of *L. amazonensis* infection, confirming a role of IGF-I in the first events of this infection in human macrophages. These findings indicate genetic advantage of this IGHD cohort against at least this particular pathogen, and confirm immune benefits of this endocrine deficiency.

## Data Availability

All datasets generated for this study are included in the manuscript and/or the supplementary files.

## Ethics Statement

The manuscript includes human studies. The local Ethical Committee of the Federal University of Sergipe approval was received for the studies (CAAE 0152.0.107.000-07), and the informed consent of all participating subjects or their legal guardians was obtained.

## Author Contributions

MB, VC, and LO performed the majority of the experiments. VC, MA-O, and RSa are endocrinologists and assisted the IGHD individuals, helped to recruit them for this study, and discussed the experimental design with the immunology group. MA, RSi, and AB helped with the macrophage infection experiments. MS helped in the Luminex experiments and statistical analysis. NP, RC, HG, RA, and AJ helped in the experimental design. MB and VC draft the manuscript. NP, MA-O, RSa, and AJ revised the manuscript. AJ was also responsible for the grants to perform this study and is the advisor of MB and LO, during Ph.D. and Masters, respectively. MA-O is the advisor of VC, during Ph.D.

### Conflict of Interest Statement

The authors declare that the research was conducted in the absence of any commercial or financial relationships that could be construed as a potential conflict of interest.
